# Identification of a Novel Tumor-Binding Peptide for Lung Cancer Through *in-vitro *Panning

**Published:** 2018

**Authors:** Babak Bakhshinejad, Habib Nasiri

**Affiliations:** a *Department of Genetics, Faculty of Biological Sciences, Tarbiat Modares University, Tehran, Iran.*; b *Department of Medical Genetics, Nika Center of Health Promotion and Preventive Medicine, Tehran, Iran.*

**Keywords:** Phage display, Peptide library, Non-small cell lung cancer, Targeted therapy, Panning, Delivery

## Abstract

Tumor-targeted therapies are playing growing roles in cancer research. The exploitation of these powerful therapeutic modalities largely depends on the discovery of tumor-targeting ligands. Phage display has proven a promising high throughput screening tool for the identification of novel specific peptides with high binding affinity to cancer cells. In the present study, we describe the use of phage display to isolate peptide ligands binding specifically to human lung cancer cells. Towards this goal, we screened a phage display library of 7-mer random peptides *in-vitro* on non-small cell lung carcinoma (A549) as the target cell. Following selection rounds, there was a highly considerable enrichment of lung cancer-binding phages and a significant increase – 170 fold - of the phage recovery efficiency. After three rounds of *in-vitro *panning, a group of peptides with different frequencies were obtained. The binding efficiency and selectivity of these peptides for target and control cells were studied. The results of cellular binding assay and cell ELISA (enzyme-linked immunosorbent assay) revealed that LCP1 (Lung Cancer Peptide1) with the displayed sequence AWRTHTP is the most effective peptide in binding to lung cancer cells compared with normal lung epithelial cells and different non-lung tumor cells. In conclusion, our findings suggest that LCP1 may represent a novel peptide that binds specifically to lung cancer cells and further studies can pave the way for its application as a potential targeting moiety in the targeted delivery of diagnostic and therapeutic agents into lung malignant cells.

## Introduction

Lung cancer is of one the most leading causes of cancer-associated mortality across the world with nearly 1.4 million deaths per year. Also, 1.6 million new cases of lung cancer are diagnosed each year (1). There are two major pathological types of lung cancer: small cell lung cancer (SCLC) and non-small cell lung cancer (NSCLC). NSCLC is responsible for approximately 85% of all diagnosed cases of the disease (2, 3).Conventional chemotherapy has proven efficacy in preventing tumor recurrence and prolonging patients overall survival. However, the lack of tumor-specific delivery of chemotherapeutic agents results in limited efficiency, increased toxicity, and severe side effects (4). Therefore, novel strategies for the selective delivery of anticancer drugs to lung malignant cells are highly desired. 

During tumorigenesis, a huge number of genetic, epigenetic, and proteomic changes occur in the cell. These changes cause extensive alteration of the cell surface characteristics, which may serve for targeted delivery of drugs to the desired tumor cell types (5). One important component of a tumor-targeted drug delivery system is a tumor-targeting ligand. Antibodies have been the focus of much investigation for the discovery and development of tumor-targeting agents. However, they have high production costs, are immunogenic, and too large to efficiently pass through the tumor tissue (6). Peptides offer many advantages over antibodies for cancer drug delivery purposes. They can be easily synthesized in large quantities, are amenable to derivatization, and can undergo a variety of chemical modifications to alter their different physiochemical properties including affinity, charge, hydrophobicity, solubility, and stability. Importantly, their much smaller size allows them to effectively penetrate tumors (7, 8). 

Phage display is a powerful high throughput screening methodology for the identification of peptides that specifically bind to different targets. This approach has become a standard tool for basic research, drug discovery, drug delivery, and pharmaceutical biotechnology. In phage display libraries, a large number of randomized peptide sequences are expressed on the phage surface as fusion to one of the phage coat proteins. These libraries can be screened through an affinity-based selection strategy called panning against target cells with resultant isolation of peptides indicating high affinity and specificity towards the cell of interest (9, 10). To date, the screening of phage display combinatorial libraries has been applied successfully to the selection of numerous cell-homing peptides, which show specific binding to different malignancies including breast (11), prostate (12), colon (13), brain (14), bone (15), ovary (16), and esophagus (17) cancer cells. 

In the present study, using *in-vitro* phage display screening we aimed to identify novel specific peptides that could bind to human lung adenocarcinoma cell line A549. Our selection strategy led to the isolation of a phage clone displaying the peptide sequence AWRTHTP that bound to A549 in a cell-specific manner. Such cancer-targeting peptides represent potential for the development of efficient platforms in the diagnosis and treatment of lung cancer.

## Experimental

*Materials*

Dulbecco’s Modified Eagle’s Medium (DMEM), Ham’s F12 medium (F12 nutrient medium), and Fetal Bovine Serum (FBS) were purchased from Gibco (USA). Penicillin, streptomycin, L-glutamine, and TMB (3,3ʹ,5,5)ʹ-tetramethylbenzidine) substrate were obtained from Invitrogen (USA). The Ph.D.^TM^-7 phage display peptide library kit was purchased from New England BioLabs (MA, USA).IPTG (isopropyl β-D-thiogalactoside) and X-gal (5-bromo-4-chloro-3-indolylβ-D-galactoside) were obtained from Appli Chem (Germany). Taq DNA polymerase 2X Master Mix Red was purchased from Amplicon (Denmark). GeneAll^® ^Expin^TM ^GelSV Kit was purchased from GeneAll Biotechnology Co. (South Korea). Mouse anti-M13 phage antibody and horse radish peroxidase (HRP)-conjugated rabbit anti-mouse secondary antibody were purchased from Abcam Inc (MA, USA). All chemicals, buffers, and bacterial culture media were obtained from Merck (USA). 

*Cell culture*

Human lung adenocarcinoma cell line A549, human hepatocellular carcinoma cell line Huh-7, human esophageal squamous cell carcinoma cell line KYSE-30, human breast adenocarcinoma cell line MCF-7, human normal lung epithelial cells SAEC, and human normal fibroblast cells were used in the study. All cell lines were purchased from Cell Bank of Pasteur Institute (Tehran, Iran) except for SAEC that was a gift from Zanjan University. Tumor cell lines were maintained in DMEM supplemented with 10% FBS, penicillin (100 U/mL), streptomycin (100 mg/mL), and 2 mM L-glutamine. Normal fibroblast cells -used for the depletion of the randomized peptide library– and normal lung epithelial cells were grown in a medium containing 1:1 mixture of DMEM and Ham’s F12 medium supplemented with 10% FBS, growth factors, and penicillin/streptomycin antibiotics. All cells were grown in 25 cm^2^ polystyrene culture flasks or six-well culture plates until they reached sub-confluent monolayers. The cells in culture were maintained at 37 °C in a humidified atmosphere of 95% air and 5% CO_2_. For routine maintenance, cells were passaged by trypsinization before becoming fully confluent. None of the cell types were retained in continuous culture for more than 1 month.

*Phage display library*

The Ph.D.^TM^-7 phage display peptide library kit contains random seven amino acid peptides fused in-frame to the N-terminus of the minor coat protein (pIII) of the filamentous M13KE phage. Therefore, 3-5 copies of a foreign peptide are expressed as part of the minor coat protein on the surface of each phage particle. The library titer is 1×10^13^pfu/mL (plaque forming units). The library has a complexity of 1×10^9^ individual phage clones. This level of complexity represents approximately all possible 7-mer peptide sequences that can be expressed by a random heptapeptide sequence. Extensive sequencing has revealed the presence of a huge diversity of displayed peptide variants with no considerable positional bias in the naïve library. The Escherichia coli strain ER2738 which is a robust F^+^ strain with a rapid growth rate and particularly well-suited for M13KE amplification was used for phage propagation. 

**Table 1:** Progressive enrichment of phages with selection rounds The phage recovery efficiency of each round was obtained via dividing the output number (the number of recovered phages) by input number (the number of phages added to the cultured cell

**Fold Increase**	**Recovery Efficiency **	**Output Number (pfu)**	**Input Number (pfu)**	**Round of Panning**
17.21	3.4 × 10^-7^	3.4 × 10^4^	1 × 10^11^	1
9.88	5.85 × 10^-6^	5.85 × 10^5^	1 × 10^11^	2
170.03	5.782 × 10^-5^	5.782 × 10^6^	1 × 10^11^	3

**Table 2:** Amino acid sequences of the peptides displayed by phages identified after three rounds of panning of Ph.D.^TM^-7 library on A549 cells

**Frequency Percent**	**Frequency**	**Peptide Sequence**	**Peptide Name**	**Phage Name**	**Phage Clone**
42	5	AWRTHTP	LCP1	P1	PC1, PC3, PC7, PC8, PC11
17	2	THSNLSV	LCP2	P2	PC2, PC10
17	2	AFRDPLY	LCP3	P3	PC4, PC6
8	1	THLSVNK	LCP4	P4	PC5
8	1	NGAYRAI	LCP5	P5	PC9
8	1	LEQTPMF	LCP6	P6	PC12

**Table 3:** Multiple alignment of peptide sequences Consensus motifs shared by different peptides are highlighted with grey color

**Sequence**							**Peptide Name**
V	S	L	N	S	H	T	LCP2
K	N	V	S	L	H	T	LCP4
F	M	P	T	Q	E	L	LCP6
Y	L	P	D	R	F	A	LCP3
P	T	H	T	R	W	A	LCP1
I	A	R	Y	A	G	N	LCP5

**Figure 1:**
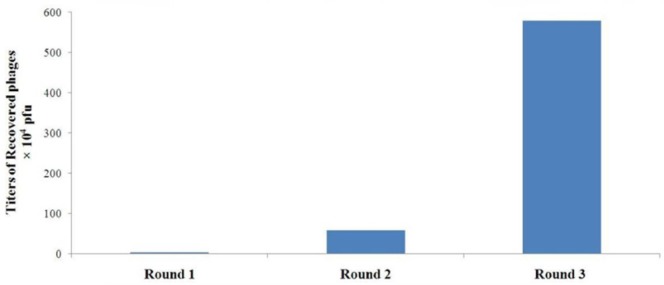
Specific enrichment of A549 cell-binding phages during rounds of *in-vitr*o panning The titers of the recovered phages from each round were determined by blue plaque-forming assay on IPTG-Xgal agar plates containing tetracycline

**Figure 2:**
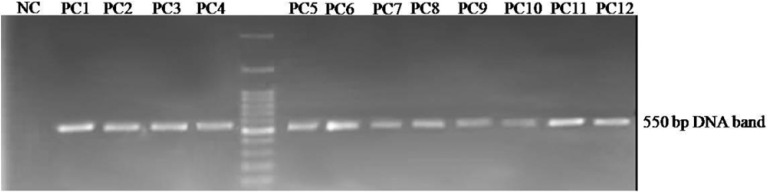
Agarose gel electrophoresis of the displayed peptide-encoding DNA insert obtained from selected phagesAn approximately 550 bp fragment is amplified by PCR on the isolated phage genomes. PC1 to PC12: Phage plaques selected from the third round of panning, NC: negative control.

**Figure 3:**
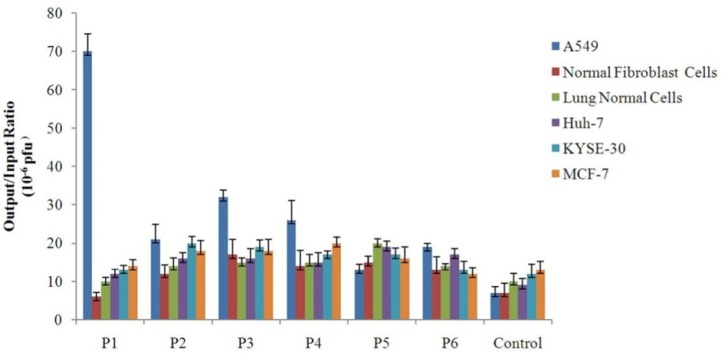
Binding of the selected phage clones to different cell types Each cell was grown to sub-confluent monolayer and then incubated individually with 10^9^ pfu of different selected phage clones. The unbound phages were washed away. Afterwards, the cell-bound phages were collected by using a low-pH buffer, and quantitated through infection of bacterial cells and titering by blue plaque-forming assay. Binding efficiency of each clone to each cell type was determined by calculating the output/input ratio. A phage with an unrelated displayed peptide was used as control for cell binding assay

**Figure 4.**
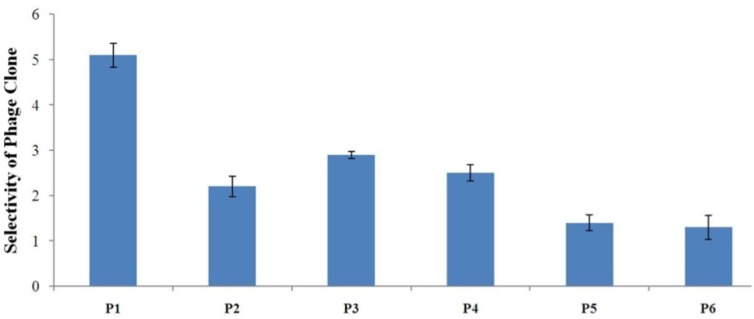
Evaluation of the binding selectivity of the selected phage clones by cell ELISA Cells were seeded onto 96-well cell culture plates overnight (1×10^4^ cells/well). 10^9^ phage particles were added to each well. Phage binding to cells was detected through adding mouse anti-M13 phage antibody and HRP-conjugated rabbit anti-mouse secondary antibody. OD was obtained after blocking the reaction. The selectivity values for phage binding was calculated by the formula mentioned in the text and were 5.1, 2.2, 2.9, 2.5, 1.4, and 1.3 for P1, P2, P3, P4, P5, and P6, respectively. P1 is indicated to be the strongest binder and binds more effectively than all phage clones to A549 cells

*In-vitro screening of phage peptide library*

A modified subtraction and selection protocol was optimized and used for the screening of the Ph.D.^TM^-7 phage display peptide library (18, 19). A549 was taken as the target cell for positive selection and normal fibroblast and Huh-7 were taken as the absorber or control cells for subtractive panning. Briefly, cells were seeded onto six-well plates and cultured in DMEM medium containing 10% FBS at 37 °C in a humidified atmosphere with 5% CO_2_.In the first round of panning, a rigorous library depletion procedure was performed in which the naïve library was successively subjected to empty multi-well plates, serum, and absorber cells (fibroblast and Huh-7 cells) before being incubated with target A549 cells. To do this depletion plan, an aliquot of the library mixed with blocking buffer was serially incubated with depleting agents for 1 h at 37 °C. Blocking buffer consisted of 2% BSA in phosphate buffered saline (PBS). In each step of the depletion screening, supernatant was removed from the well and used for the following depletion step. In the second and third rounds of selection, only control cells were exploited for subtractive panning. 

During rounds of panning, cells were washed with PBS and kept in serum-free medium for 1 h at 37 °C. Before phage application, the cells were blocked with blocking buffer for 1 h. Then, an aliquot of the library that contained 10^11^pfu was added to control cells and incubated for 1 h with gentle shaking. The non-binding phages were removed and added to target A549 cells.After incubation for 1 h, the unbound or weakly-bound phages were wiped off by extensive washing three times with PBS and five times with tris buffered saline containing tween 20 (TBST). The cell surface-bound phages were recovered by treating A549 cells with elution buffer (0.2 M glycine/HCl, pH = 2.2) for 10 min on ice and then neutralizing with 1M Tris-HCl. A small amount of the recovered phages was used for titering through blue plaque-forming assay on LB agar plates containing IPTG and X-gal. The remaining phages were amplified by infecting with ER2738 bacterial culture to be used as input for the next round of *in-vitro* selection. During rounds of selection, panning intensity was progressively enhanced by increasing the number of washing times with PBS and TBST from 8 for the first round to 12 for the last round. 

*Amplification of phage clones*

ER2738 strain was inoculated into LB medium to achieve log-phase bacterial culture. The phage suspension recovered from each round of panning was added to 20-25 mL of LB medium containing ER2738 and the mixture was incubated at 37 °C with shaking for 4.5-5 h. Following precipitation of bacterial cells by centrifugation, 1/6 volume of 20% polyethylene glycol (PEG)/2.5M NaCl was added to the recovered supernatant and incubated at 4 °C overnight or on ice for 2 h. After two successive PEG/NaCl precipitation steps, phage particles were harvested by centrifugation at 13000 rpm for 20 min. The resulting phage pellet was resuspended in tris buffered saline (TBS). 

*Phage titering*

To determine the phage titer, phages were serially diluted in liquid LB and each diluted solution was incubated with a log-phase culture of ER2738 for 5-10 min at room temperature to allow infection of bacterial cells by phage particles. 2-3 mL of melted top agar (45 °C) was added and the infected bacteria were spread on LB agar plates containing IPTG (50mg/mL) and Xgal (40 mg/mL). Plates were incubated overnight at 37 °C. Finally, the titer of the phage solution was calculated by counting the number of blue plaques appeared on the plate after 12-16 h and using the following formula:

Phage titer (pfu/mL)=a ×b ×1000 μLd

Where *a* is the number of blue plaques, *b *is the times of dilution, and *d *is the plated volume of the phage suspension (μL)

*PCR amplification and DNA sequencing of selected phages*

Plaque PCR was performed to amplify the inserts encoding peptides displayed on the surface of the selected phages. A portion of an individual well-isolated plaque was scraped up by using a pipette tip and then dipped into a PCR tube containing 25 μL of Taq DNA polymerase 2X master mix red, 2.5 μL of each of the forward and reverse primers (20 mM), and 20 μLof deionized water to reach the total reaction volume of 50 μL. Taq DNA polymerase 2X master mix red consists of the following components: 0.2 units/μL of Taq DNA polymerase, the NH_4_^+^ buffer system, 0.4 mM of each dNTP, and 1.5 mM MgCl_2_. The sequences of the forward and reverse primers were as follows: Forward primer:5ʹ-TTTAGTCCTCAAAGCCTCTG-3ʹ and Reverse primer: 5ʹ-CAAGCCCAATAGGAACCC- 3ʹ. The primers were designed by using Oligo Primer Analysis Software v.7 The phage DNA was amplified by using the following PCR program in a thermal cycler: 30 cycles (30 sec at 94 °C, 30 sec at 60 °C, and 30 sec at 72 °C) preceded by an initial denaturation for 5 min at 95 °C and followed by a final extension for 10 min at 72 °C. The amplified DNA was purified from gel by using GeneAll^®^Expin^TM^GelSV Kit and finally sequenced by ABI 3700 (Applied Biosystems, USA). 

*In-vitro cellular binding efficiency of phage clones*

A549, Huh-7, KYSE-30, MCF-7, normal lung epithelial cells, and normal fibroblasts were used for cellular binding assays. Briefly, 1×10^4^ cells were grown in each well of a 96-well cell culture plate for 24h to reach sub-confluent monolayers. Cells were then incubated with serum-free medium for 1h.1×10^9^pfu of each phage clone was added separately to different cell types and incubated for 1h at 37 °C with gentle agitation every 5 min. The unbound phages were carefully removed from the wells and the cells were washed 6-8 times with cold washing buffer (3-5 min for each time). Cell-associated phages were collected by adding 100 μL of an acidic elution buffer for 10-15 min on ice and then neutralizing with 100 μL of 1M Tris-HCl. The retrieved cell-bound phages were titered by infecting bacteria and phage binding efficiency was calculated through dividing the number of output (recovered) phages by the number of input phages. Phage displaying an unrelated peptide was used as control. 

*Cell ELISA with phage*

Cells were cultured in DMEM and seeded onto 96-well plates (1×10^4^ cells/well) the day before use. Cells were then incubated with serum-free medium at 37 °C for 1h and fixed by using ice-cold 4% paraformaldehyde (PFA) for 10-15 min. The fixed cells were washed with PBST (PBS containing 0.05% w/v Tween 20) and blocked with 100-200 μL of blocking buffer at 37 °C for 1.5-2 h. Phage clones were added into wells (1×10^9^pfu/well) and the plates were kept at 37 °C for 1h. Subsequently, the unbound phages were removed by washing three times with TBST. To detect the bound phages, cells were incubated at 37 °C for 1h with 100 μL/well of mouse anti-M13 phage antibody, washed three times with PBST, and then incubated with 100 μL/well of HRP-conjugated rabbit anti-mouse secondary antibody. After washing, color development was carried out by adding 100 μL of freshly prepared TMB substrate to each well and incubating the plate in the dark for 5 min. The reaction was terminated via adding 50-100 μL of 0.5 M H_2_SO_4_ and the absorbance (OD) values were measured by an automated ELISA plate reader (BioTek Instruments Inc, USA) at wavelength of 450 nm. Selectivity of phage clones was calculated by using the following formula (20); 

$\mathrm{Selectivity}=\frac{\mathrm{S1}-\mathrm{C1}}{\mathrm{S2}-\mathrm{C2}}$

whereS1 and C1 represent the OD values obtained from binding of the selected phage and control phage to A549 cells and S2 and C2 represent the OD values obtained from binding of the selected phage and control phage to control cells.

*Statistical analysis*

All statistical analyses for *in-vitro* cellular binding assay and cell ELISA were performed by using Microsoft Office Excel 2007 and Graph Pad Prism 5. Statistical differences among samples were evaluated by one-way ANOVA and *P*< 0.05 was considered as statistically significant. 

## Results

*Specific enrichment of phage clones that bind to A549 lung cancer cells*

The Ph.D.^TM^-7 phage display library that is based on filamentous M13 phage as a vector was screened for the selection of peptide ligands binding to the surface of A549 cells. Phage clones bound specifically to human lung tumor cells were identified by three rounds of positive panning and three rounds of subtractive (negative) panning *in-vitro. *Subtractions were performed to eliminate potential nonspecific-binding phage clones. 

Our results showed that after each round of *in-vitro* selection both the titer of recovered phages and recovery efficiency are enhanced. Following the third round of selection, there was an approximately 170-fold increase in the number of phages recovered from A549 lung cancer cells compared with the first round (Figure 1). In contrast, there was a decrease in the number of phages retrieved from control cells. Furthermore, the ratio of output to input phage number after each round of selection was used to determine the recovery efficiency. The results indicated the increase of the phage recovery efficiency from 3.4×10^-7^ to 5.782×10^-5 ^(Table 1). These observations provide convincing evidence for the successful selection and effective enrichment of phage clones that specifically bind to A549 lung cancer cells.

*PCR amplification of the peptide-encoding sequences in lung cancer cell-bound phages*

PCR was performed to amplify the displayed peptide-encoding sequences in the genome of phages recovered from the last round of panning and then plated out on agar containing IPTG-Xgal. Amplified fragments of the phage genomes approximately 550 bp in size were separated via electrophoresis on 1% agarose gel. The amplified DNA was visualized by a gel documentation system under UV light (Figure 2). All amplified PCR products were finally purified from gel and sequenced.

*DNA sequencing of the peptide sequences bound to lung cancer cells*

After *in-vitro* selection, a total of 12 phage clones were randomly picked out from the output of the final round of panning for sequencing and further analysis. The peptide-encoding DNA inserts in the genomes of selected plaques were amplified by PCR, sequences of the inserts encoding displayed peptides were determined by phage DNA sequencing and translated by Translate tool in ExPASy bioinformatics resource portal (http://web.expasy.org/translate). The translation of foreign oligonucleotide inserts in the phage DNA revealed displayed peptide sequences responsible for phage binding to A549 lung tumor cells. 

Table 2 summarizes the amino acid sequences of the displayed peptides encoded by DNA inserts in the selected phage clones. Each of the phage clones as well as corresponding exogenous peptide sequences was given a sequential name from P1 to P6 and from LCP1 to LCP6 (LCP is the acronym of Lung Cancer Peptide), respectively. Sequencing of the phage clones demonstrated that the procedure of cell panning has led to the enrichment of six unique peptide sequences. Among the isolated peptides, LCP1 clone was the most dominant and appeared most frequently. This peptide was found in 42 percent (5 out of 12) of the sequenced plaques. Each of the sequences designated LCP2 and LCP3 represented two of the clones and each of the peptides LCP4, LCP5, and LCP6 appeared only once.

Multiple sequence alignment of the peptide sequences was performed by using Lasergene (v7.1) and MUSCLE (EMBL-EBI, http://www.ebi.ac.uk/Tools/msa/muscle) programs to determine the groups of related peptides. Some sequence similarities were observed among isolated peptides. Multiple sequence alignment analysis revealed that three of peptides (LCP2 and LCP4) contained the consensus tripeptide motif LSV, eight of peptides (LCP1, LCP2, and LCP4) contained the consensus motif TH, and eight of peptides (LCP1, LCP3, and LCP5) contained the non-continuous motif AXR (where X represents an aromatic amino acid). Also, the common dipeptide motif TP was present in six of peptides. All consensus amino acid motifs are indicated as grey in Table 3.

*Binding efficiency of the isolated phage clones*

*In-vitro* cellular binding assay was used to measure the binding efficiency of the selected phage clones to different cell types that is defined as the ratio of output phage to input phage. In addition to cell types used for screening procedure, normal lung epithelial cells, KYSE-30, and MCF-7 were also incorporated into cell binding experiment. The results of cellular binding assay indicated among all of the isolated phages the clone P1 has the highest binding efficiency to A549 cells when compared with other cell types. Furthermore, although the association of P3 and P4 clones with A549 cells was weaker than P1, they showed stronger binding to A549 cells than control cells. P2 was not an efficient specific phage because it showed relatively similar binding to lung cancer and control cells in particular non-lung cancer cells. P5 and P6 were weak binders with the lowest output/input ratio. The isolation of clones such as P5 and P6 with negligible difference in binding to target and different control cells of different tissue origins may be ascribed to their binding to common receptors on the surface of these cells. Such clones could be misleading and distance us from the goal of identifying cell-specific peptide ligands. The results provided strong support for the notion that binding of the clone P1 to A549 lung cancer cells is specific.

Also, Figure 3 indicates that binding of most clones to KYSE-30 and MCF-7 cells - which were not used in subtractive panning - is higher than control cells used in subtractive panning. This implies the fact that exploitation of a larger number of cell types as control in subtractions may lead to the more effective elimination of nonspecific phages and isolation of ligands with higher binding specificity.

*Binding selectivity of the isolated phage clones by cell-based ELISA*

To determine binding selectivity of the isolated phages to lung cancer cells, cellular ELISA was performed on phage clones identified after the third round of *in-vitro* panning (Figure 4). To calculate selectivity, binding of each phage clone to A549 cells was compared with binding to normal lung epithelial cells. The results of ELISA showed the clone P1 clone has the highest binding selectivity to A549 cells when compared with other phage clones. Therefore, the sequence AWRTHTP seems to be the strongest binding peptide to lung cancer cells. P3 was shown to be a relatively good binder. Binding to A549 cells of P5 and P6 was not considerably high and their selectivity values were very low. These clones may be considered as non-specific binders.

## Discussion

One of the major challenges of cancer therapy is the lack of selectivity of therapeutics that leads to high toxicity of curative approaches in clinical oncology. The development of improved strategies for targeted delivery of therapeutic compounds to malignant cells and tissues provides an ideal means to maximize the antitumor efficacy of drugs and minimize their adverse effects. Tumor cells typically present vast numbers of distinctive cell surface molecules such as tumor-associated antigens (TAAs) or tumor-specific antigens (TSAs)(21). These surface molecular markers could serve as potential portals for the delivery of pharmaceutical agents into the diseased cells. Within the recent years, phage display has shown huge potential for the selection of novel targeting ligands with desired binding towards different tumor cells (22, 23). In the present study, a 7-mer peptide phage display library was screened *in-vitro* and phage clones bearing peptide sequences that could potentially bind to the surface of A549 human lung cancer cells with high binding efficiency and selectivity were successfully isolated.

To deplete the library of non-specific binders, we used a modified screening procedure. Our optimized protocol had several features that decreased background binding and significantly improved the chance of obtaining specific phages with high binding selectivity towards lung cancer cells. These features included several rounds of whole-cell subtractive panning on normal and tumor control cells, subtractive panning on polystyrene cell culture plates and bovine serum albumin (BSA), and extensive washing in which the number of washing steps and incubation times gradually increased with the progress of rounds. These measures enhanced stringency and selection pressure of the panning scheme in favor of the enrichment of target-specific phages. Titering the input and output phage exhibited a dramatically progressive enhancement of the phage recovery rate at the end of panning rounds, which is the preliminary evidence of the effective enrichment and selection of phages with binding capacity to A549 cells. Our selection scheme also led to the isolation of a limited number of peptide sequences (six unique peptides) among which the sequence AWRTHTP had a higher frequency than other peptides (5 out of 12 peptides). Furthermore, one consensus tripeptide motif and several consensus dipeptide motifs were observed in the different isolated peptides. The presence of consensus amino acid motifs in different sequence contexts and the appearance of peptide sequences with high frequency after rounds of panning highlight the fact that our phage display selection has proven successful in identifying lung cancer cell-binding ligands

(24, 25). 

Bioinformatics analysis through PhD Faster tool (26) in SAROTUP (Scanner and Reporter Of Target Unrelated Peptides) database as the most extensive bioinformatics source for evaluating peptides derived from phage display selections (27) suggested that AWRTHTP is not a target-unrelated peptide (TUP). TUPs are non-specific peptides that can mislead the results of phage display screenings (28, 29). PhD Faster is a strong bioinformatics tool by which TUPs can be identified. However, analysis by Mimo Blast(30) in the SAROTUP suite indicated that the clone P6 displaying the peptide LEQTPMF has been isolated previously through panning of Ph.D.^TM^-7 phage display library on an inorganic target called crystalline Ni3B (31). The MimoBlast tool in the SAROTUP suite is developed to evaluate if there are peptides that are identical or similar to new peptides identified in panning experiments. The Mimo Blast analysis leads us to the conclusion that although LEQTPMF has been identified in our phage display selection, it could not be a specific binder to lung cancer cells. The isolation of this peptide on two targets with different identities suggests its potential to be a target-unrelated peptide; a conclusion that warrants more investigation to be confirmed. 

In screening protocol, subtractions were performed on human normal lung fibroblast cells and Huh-7 (as a tumor cell of non-lung origin). Fibroblasts are widely distributed in the body and could be an appropriate representative of normal non-malignant cells. Also, the exploitation of Huh-7 as a cell type of hepatic origin highly eliminates the possibility of non-specific capture of phage particles (32, 33). However, for future selections the use of a wider variety of control cells in panning procedure could significantly improve the isolation of target-binding ligands. 

Further characterization of the selected phage clones through *in-vitro* cellular binding assay and cell-based ELISA confirmed the high binding affinity and selectivity of the clone P1 displaying AWRTHTP towards A549 cells. The results of both tests revealed that the phage with AWRTHTP displayed on its surface is the best binding clone compared with all other selected phage clones. Interestingly, these findings agreed well with the outcome of panning procedure; the phage clone with the highest frequency in panning was the best binder in cell-based assays. The correlation between peptide frequency and its binding strength to the target cell provides strong and meaningful support for the success of our panning procedure in isolating a lung cancer-specific peptide. Further studies are needed to determine the cellular receptor to which the peptide AWRTHTP binds. Identification of this receptor will help us in unraveling the mode of action of the peptide and accelerate its potential translation into the clinic. But, it should be kept in mind that although receptor identification offers biologically significant information, the peptide AWRTHTP can be used for targeted gene and drug delivery purposes without knowledge of its interaction with the related cellular receptor. 

Compared with *in-vivo* phage display technology, *in-vitro *cell-based panning is both simple and effective. Whole intact cells mimic the normal physiological conformation of cellular receptors and present an antigenic surface repertoire that is as similar as possible to the situation encountered *in-vivo*. In this context, selection occurs in a more relevant cellular context, while the cell surface topography is preserved (34). Also, it has been demonstrated in *in-vivo *phage display selections that phage particles can be absorbed largely by the reticuloendothelial system present in organs such as liver before reaching target tumor tissues (35, 36). This hepatic uptake is followed by rapid clearance of phage particles from the blood and the whole body. Also, *in-vivo* selection is highly dependent on the vascularization of the target organ. The capillary vessels of the vascular system can represent an important and intractable barrier for phage particles to pass through; therefore the great majority of the recovered phages are actually binding to the endothelial cells of the vasculature not tumor cells. This reveals that most phages are cleared through adhering to endothelial cells before reaching the tumor site of interest. There are several reports confirming that *in-vivo* phage display has led to the isolation of vascular endothelium-binding peptides (37-39). 

Our study demonstrated specific binding of the peptide AWRTHTP to human lung cancer cell line A549. However, for clinical applications more comprehensive studies are required to fully examine the lung cancer specificity and selectivity of the peptide by using tissue samples extracted directly from patients and *in-vivo* animal models with xenografts lung tumors. The peptide AWRTHTP could add to the growing toolbox of tumor targeting agents. The identification of peptides such as AWRTHTP presents the possibility of coupling antitumor drugs directly to the peptide for targeting lung tumor cells. Also, it can be coupled to the surface of gene transfer vectors and drug delivery vehicles and used for the development of improved gene and drug delivery systems for lung cancer therapy. 
